# Effects of an ageing population and the replacement of immune birth cohorts on the burden of hepatitis A in the Netherlands

**DOI:** 10.1186/1471-2334-13-120

**Published:** 2013-03-05

**Authors:** Scott A McDonald, Marie-Josée J Mangen, Anita Suijkerbuijk, Edoardo Colzani, Mirjam EE Kretzschmar

**Affiliations:** 1 Centre for Infectious Disease Control, National Institute for Public Health and the Environment, Bilthoven, The Netherlands; 2 Julius Centre for Health Sciences & Primary Care, University Medical Centre Utrecht, Utrecht, The Netherlands; 3 Centre for Nutrition, Prevention and Health Services, National Institute for Public Health and the Environment, Bilthoven, The Netherlands; 4 European Centre for Disease Prevention and Control, Stockholm, Sweden

**Keywords:** Hepatitis A virus, Dynamic transmission model, Disability-adjusted life-years, Natural immunity, Population ageing

## Abstract

**Background:**

In populations in which the incidence of hepatitis A virus (HAV) infection has declined due to socio-economic improvements, better sanitation and hygiene, and vaccination, birth cohorts who have long-term immunity through exposure early in life are now being replaced by non-immune cohorts, meaning that more cases in the elderly may occur in future. Our goal was to qualitatively investigate the interaction of this cohort effect and demographic change (population ageing) on the estimated disease burden of HAV infection in the Netherlands.

**Methods:**

We used dynamic MSIR (maternal immunity–susceptible–infectious–recovered) transmission and demographic models to simulate annual HAV incidence over the period 2000–2030, and estimated disease burden using the disability-adjusted life years (DALY) measure and a pre-defined disease progression model. Five scenarios representing different force of infection situations were simulated.

**Results:**

The overall disease burden was projected to decrease over the simulation period in the baseline scenario (310 DALYs in 2000 compared with 67 in 2030). This decreasing trend was absent for the 55+ years age group; 23.5% of all new infections were predicted to occur in the 55+ group in 2030, compared with 5.5% in the 55+ group in 2000.

**Conclusions:**

In the absence of further public health interventions and under the assumption of a continued steady decline in the force of infection, the HAV disease burden in the Netherlands is predicted to decrease over the coming decades, but with proportionally more of the burden occurring within the increasingly larger segment of the population represented by elderly persons who are no longer naturally immune.

## Background

In the Netherlands, hepatitis A virus (HAV) infection is not considered a major public health problem because of the low incidence of infection; the majority of new infections are observed in defined risk groups such as men who have sex with men (MSM) and travellers to countries where HAV is endemic. Due to improved socio-economic and sanitation conditions since the end of the Second World War and an increase in vaccine-induced protection in recent years [1], the force of infection (FOI; the risk per susceptible person per unit time of becoming infected) has steadily declined [2-4]. However, the size of the susceptible population and the likelihood of outbreaks occurring are expected to increase in the coming decades because of the cohort effect: younger birth cohorts have less exposure to HAV, and over time the segment of the population with natural HAV immunity is being replaced. Seroprevalence surveys conducted in 1995/6 and 2006/7 showed that 77% of persons in the Netherlands born before 1945 were anti-HAV positive [1,5], compared with fewer than 10% born after 1960 [5].

Dynamic demographic processes such as mortality, fertility, and migration that influence the age-distribution of the population also have an impact on the projected number of HAV cases, and consequently the estimated population-level disease burden. Complications from HAV are more frequent and more severe in adults than in children [6]; hence, any public health benefit from a decreasing trend in the force of infection may be offset by the increased morbidity and mortality expected with an older average age at infection.

In the current study we developed a simple deterministic sex- and age-structured model of the transmission dynamics of HAV, integrated with a dynamic model of demographic change. The transmission model was needed because exposure is birth cohort-dependent, under the assumption of a decreasing FOI that is attributable to improvements in hygiene. We calculated disease burden based on estimated annual incidence generated through simulation of HAV transmission over the period 2000–2030, to assess the interaction between the diminishing of natural immunity in the population (i.e., the replacement of immune cohorts) and population ageing on the burden of HAV in the Netherlands. We also compared the predicted burden associated with each of these two factors alone by removing the other from the model. Recently, HAV burden has been computed based on current notification data and estimates of under-reporting/under-ascertainment [7], but forecasts have not been made. Our aim was not to precisely estimate the current or future disease burden, but to qualitatively investigate the differences in estimated burden under various transmission scenarios.

## Methods

### Modelling infection dynamics

**Figure 1:**
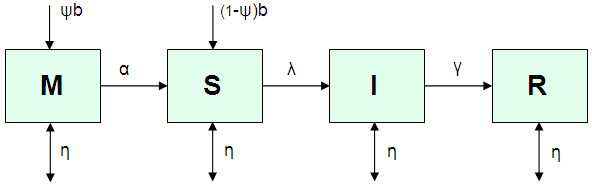
**Compartmental model for the transmission of hepatitis A, shown for the age group <1 years (for all other age-groups, the inflows to the M and S compartments due to births are not present).** The model is stratified by sex and and by age (1-year age groups, from <1 years to 85+ years), with ageing simulated by advancing all compartments one age group at the end of each simulation year.

A sex- and age-structured MSIR (maternal immunity—susceptible—infectious--recovered) deterministic model of the infection dynamics of HAV in the Netherlands population was implemented. In this approach the population is stratified into ‘compartments’ according to sex, 1-year age group (<1 years through 85+ years), and status (maternal immunity, susceptible, infected/infectious, recovered/immune) (Figure 1); flow of individuals between compartments occurs at rates defined by the model parameters and described by a set of differential equations. Ageing was modelled by advancing all compartments one age group at the end of each simulation year. The model parameters are: α (the rate of loss of maternal immunity after birth), λ (the force of infection (FOI), or the rate that susceptibles become infected), γ (the rate that infected/infectious persons recover and develop life-long immunity), b (the birth rate), and η (the rate of entering/exiting compartments due to demographic processes)(see Table 1). These processes – migration, death, and ageing, are described below. Although the actual HAV situation in the Netherlands is perhaps best characterised by individual and small clusters of cases, for convenience we model it as an endemic disease.

**Table 1:** MSIR transmission model parameters, values, and sources

**Parameter**	**Value**	**Reference**
α – rate of loss of maternal immunity	1/α = 9 months	Lieberman et al. [13]
Ψ – proportion of babies born with maternal immunity	0.13 (males) 0.17 (females)	Pienter study (de Melker et al.[11]; van den Hof et al.[12])
γ – rate of recovery from infection	1/ γ = 5 weeks	Leach [10]
ϕ – proportion of travel-related cases (used during fitting only)	0.356	van der Eerden et al. [8]
β_dom,a_*(t)* – transmission rate (domestic), *t*=2000, for age-groups {<1, 1–4, 5–9, 10–14, 15–19, 20–24, 25–29, 30–34, 35–39, 40–59, 60+}	{0.114,20.5,41.0,27.3,9.11, 0.114,0.114,0.455,6.83, 4.55,9.11}	Estimated
β_trav_*(t)* – transmission rate (travel-related), *t*=2000, age-independent	0.0000505	Estimated
λ_a_(*t*) – force of infection, age-group specific: sum of domestic and travel-related transmission: λ_dom,a_(*t*)+ λ_trav_(*t*)	$\lambda_{\mathrm{dom},a}\left(t\right)=\beta_{\mathrm{dom},a}\left(t\right)\frac{\sum_{j}I_{j}\left(t\right)}{\sum_{j}N_{j}\left(t\right)}$ *λ*_*trav*_(*t*) = *β*_*trav*_(*t*)	Srinivasa Rao et al. [18]
b – birth rate	Sex and year dependent; age group <1 only	Estimated from data held by Statistics Netherlands
η – net rate of demographic factors (mortality, immigration, emigration)	Sex, age-group, and year dependent	Estimated from data held by Statistics Netherlands

Imported (travel) cases and transmission among MSM are two important routes of HAV infection. In the period 1993–2002, notification data indicated that 64% of infections were acquired in the Netherlands, with 18% of infections acquired in Morocco and Turkey, countries where HAV is endemic [8]. Incidence rates among travellers to these countries, particularly children, dropped markedly between 2000 and 2005 [9], consistent with targetted vaccination efforts. Based on these findings, we assumed that 36% of infections were acquired through travel to an endemic country. The overall FOI (λ_a_) and domestic transmission rates (β_dom,a_) were estimated separately for each age-group (Table 1) by visually fitting the model to the age distribution of notified cases (national data from OSIRIS; data and model fit are provided in the Additional file 1: Figure A2). The transmission rate for travel-acquired infection (β_trav_) was therefore defined as 0.36 λ. The FOI for domestically acquired infections (64% of total) depends on the fraction of infectious persons in the population (see Table 1 for parameter summary).

The rate of recovery from infection (γ) was set at 10.4 year^-1^, based on an estimated mean duration of infectiousness of 5 weeks [10], and the assumption that this duration is exponentially distributed.

A large national seroprevalence study in the Netherlands (Pienter) was conducted in 1995–1996 (*n* = 7373) [11,12]. Sex- and age-group specific seroprevalence from the Pienter dataset were used as initial values for the recovered/immune model compartment. Vaccination uptake was assumed to be constant over the time period modeled; thus it does not impact on transmission dynamics.

Maternal immunity is short-lived, with antibody detected after 1 year in only 39% of babies born HAV antibody-positive [13]. The parameter Ψ represents the proportion of babies born with maternally-acquired immunity, and was estimated at 13% for males and 17% for females, based on seroprevalence survey data for the <1 year age group [11,12]. The rate of loss of maternal immunity was set to 1.33 year^-1^, derived assuming a mean duration of 9 months [13].

### Modelling demographic dynamics

Dynamic modelling of the Netherlands population was undertaken for the period 2000–2030, using demographic information available from Statistics Netherlands (CBS) for 2000, specifically population size stratified by sex and 1-year age-group [0 to 85+ years], age- and sex-specific mortality rates, age-specific fertility rates, and age-specific net migration rates. Life-table methods were used to calculate life expectancy for each age-group from mortality rates, and sex- and age-specific net migration rates were fixed as the mean net migration over the period 2000–2009 (see [14] for further details). The evolution of the age distribution of the population over the simulation period was computed using Leslie matrices [15].

### Modelling disease progression

The outcome tree for HAV disease progression was adopted from that developed as part of the Burden of Communicable Disease in Europe (BCoDE) project [16]. Although acute HAV infection can lead to complications and severe sequelae, such as fulminant hepatitis, liver failure, prolonged cholestasis, and Guillain-Barré syndrome, such outcomes are very rare and were therefore not incorporated into the disease progression model (Additional file 1: Figure A1), similar to Havelaar et al. [7]. Disability weights and durations were adapted from those compiled for the BCoDE project (see Additional file 1: Table A1 for details), which were based on Havelaar et al. [7].

The greatest risk of mortality from acute HAV infection is observed in the elderly [17]. We used published age-specific case-fatality rates to specify mortality following acute infection [4,18,19].

### DALY computations

To estimate disease burden, we used the pathogen-based approach, in which all (future) health outcomes causally related to infection with the pathogen, including acute infection, are taken into account in the calculation of the total disease burden [16,20]. We used the disability-adjusted life-years (DALY) measure [21] which quantifies the difference, in years, between ideal health and actual health status associated with illness, disease, or injury. One DALY corresponds to one lost year of healthy life, or to multiple years experienced at less than full health.

The DALY measure is the sum of two components: years of life lost (YLL) due to premature death and years of life lost due to disability (YLD). YLD is computed as the product of the disability weight and duration of illness associated with a specific health outcome, accumulated over the number of incident cases in all health outcomes including and leading from acute infection (for more details see Mangen, Plass, Havelaar, Gibbons, Cassini, Mühlberger, van Lier, Haagsma, Brooke, Lai, et al: The pathogen- and incidence-based DALY approach: a new methodology for estimating the burden of infectious diseases in Europe, Submitted). The disability weight for acute infection was estimated as the weighted average of the disability weights associated with three severity levels (hospitalised, visiting a GP, and not visiting a GP). [7] YLL is calculated as the number of deaths causally related to development of a particular health outcome multiplied by the life expectancy at the age of death, summed over all health outcomes.

Uncertainty in the model-generated incidence time-series was estimated as in Haagsma et al. [22] by assuming that the simulated annual number of cases is produced by a Poisson process; thus variability can be expressed by a Gamma distribution (with parameters shape = mean simulated annual number of cases, scale = 1) and the range of uncertainty by the 5th and 95th percentiles of this distribution. Confidence intervals were then constructed around DALYs using Latin hypercube sampling methods [23]. Demographic and transmission dynamic models were implemented using R statistical software [24].

### Scenario analyses

Five scenarios corresponding to various FOI patterns were investigated. In the baseline scenario, an exponentially decreasing FOI over time of 5% per year, but constant across sex- and age-groups was implemented by treating the transmission rate parameters β_a_ as time-varying, by multiplying the baseline value by exp(δ * (*t* – 2000)), where δ is −0.05 and *t* is year of simulation. The value for δ is consistent with the hypothesised decline in Europe since the end of the Second World War [3] and the average 4.5% decline in FOI reported for the USA [25] throughout the 20th century. Scenario 2 examined the effect on disease burden in the case of a constant transmission rate over time, to isolate the impact of the annual 5% decrease assumed in the baseline scenario, Scenarios 3–5 investigated the impact of large point outbreaks occurring in two different years (2015 and 2025) during the simulation period, by setting the transmission rate to three times the baseline value during a single calendar year. Scenario 3 simulated an outbreak in primary school-aged children 5–9 years old, the age group with largest proportion (33%) of notified cases in the Netherlands in the period 1993–1997 [26]; β was increased only for this age-group. In Scenario 4, an outbreak due to sexual transmission within MSM was simulated, by confining the three-fold increased transmission rate to men between 25–44 years of age (roughly corresponding to the age range of 25–48 years represented by a cluster of 29 HAV cases in MSM reported in Rotterdam in 1998 [27]). Scenario 5 simulated a potential outbreak in an old peoples’ home (potentially attributable to a foodborne source of infection), by restricting the three-fold increased β to persons aged 80 years and over. To adjust for differences in subpopulation sizes, the standardised measure DALYs per 100,000 was also calculated for each scenario.

## Results

### Transmission dynamics

**Figure 2:**
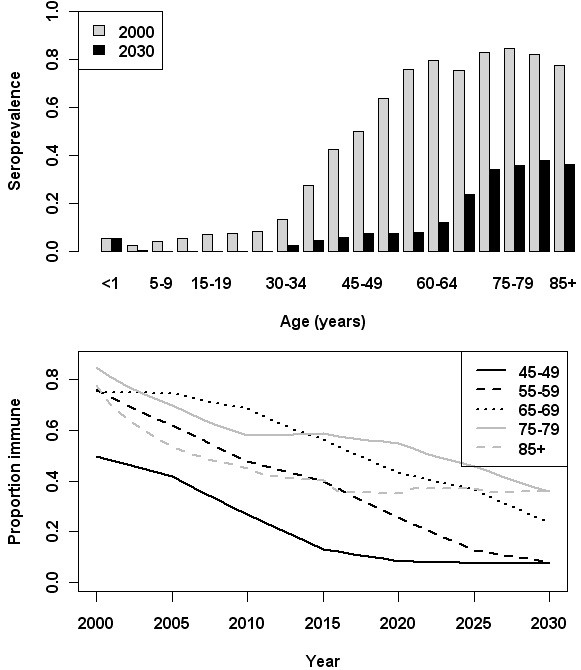
Estimated HAV seroprevalence by age group in 2000 and 2030 (upper panel); the estimated proportion of the population with HAV immunity between 2000 and 2030 for selected age groups (lower panel).

The model (baseline scenario) produced a shift in the age distribution of immunity over time, as shown by the projected seroprevalence according to age group (Figure 2); immunity (the proportion immune) decreased for all age groups over the simulation period (Figure 2).

**Figure 3:**
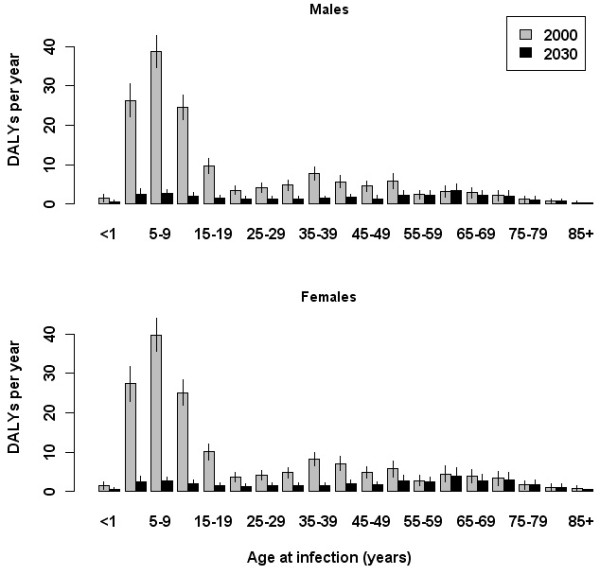
**Estimated age-group specific burden of HAV.** Acute infections occurring in the years 2000 and 2030 are compared, and are plotted separately for males (upper panel) and females (lower panel). Vertical lines indicate 95% confidence intervals.

The estimated overall number of new infections in the year 2000 was 1,547, corresponding to an estimated incidence of 9.8 per 100,000 population. The age-group with the largest proportion of new infections (27.8%) was 5–9 years. The age distribution was less skewed by the end of the simulation period; in 2030 the largest proportion of new infections (10.6%) was also predicted to occur in the 5–9 years age-group, with relatively more infections occurring in the older age groups compared with 2000 (Figure 3). 23.5% of all new infections in 2030 were represented by persons 55 years and older, compared with 5.5% in 2000. Age-group specific incidence estimated for the years 2000 and 2030 is provided in Table 2.

**Table 2:** Modelled incidence (acute infections per 100,000) by age-group in the years 2000 and 2030

**Age group (years)**	**Incidence in 2000 (95% CI)**	**Incidence in 2030 (95% CI)**
<1	5.1 (2.5–7.9)	1.8 (0.48–3.1)
1-4	24.4 (21.1–27.6)	2.1 (1.2–3.0)
5-9	43.1 (39.3–47.1)	2.8 (1.9–3.8)
10-14	29.9 (26.7–33.1)	2.4 (1.5–3.2)
15-19	13.0 (10.7–15.2)	1.8 (1.0–2.5)
20-24	4.8 ( 3.6–6.0)	1.5 (0.89–2.2)
25-29	4.7 (3.6–5.9)	1.6 (0.93–2.2)
30-34	4.8 (3.7–5.8)	1.6 (0.89–2.2)
35-39	8.5 (7.1–9.9)	1.7 (1.1–2.4)
40-44	5.5 (4.3–6.7)	1.6 (0.97–2.3)
45-49	4.8 (3.7–6.0)	1.6 (0.90–2.2)
50-54	3.5 (2.5–4.4)	1.5 (0.79–2.1)
55-59	2.3 (1.4–3.3)	1.4 (0.79–2.0)
60-64	2.8 (1.7–3.9)	1.4 (0.80–1.9)
65-69	3.4 (2.1–4.6)	1.1 (0.59–1.6)
70-74	2.3 (1.2–3.4)	0.88 (0.39–1.3)
75-79	1.8 (0.87–2.9)	0.72 (0.27–1.2)
80-84	1.9 (0.68–3.2)	0.50 (0.15–0.87)
85+	1.9 (0.56 –3.2)	0.34 (0.08–0.60)

### Population dynamics

The demographic model projected steady growth in the size of the Netherlands population, from 15.9 million persons in 2000 to 17.6 million in 2030, with an increase in the proportion of 55–74 year-olds and 75-plussers from 18% and 6%, respectively, to 25% and 23%, and a decrease in the proportion of 35–54 year olds, from 30% to 23%, over the same period.

### Estimated future burden from HAV

A five-fold decrease in the overall annual disease burden of HAV in the Netherlands was predicted over the simulation period, from 310 DALYs (95% CI: 296–326; YLL = 231, 95% CI: 221–244) in the year 2000 to 67 DALYs (95% CI: 60–75; YLL = 54, 95% CI: 48–60) in 2030. This decrease was mostly confined to persons younger than 55 years (Figure 3).

**Figure 4:**
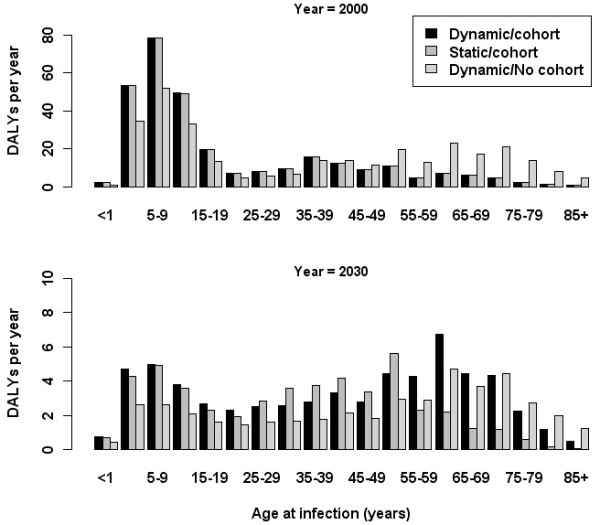
**Comparison of assumptions with respect to the dynamic modelling of demography and the cohort effect.** The estimated annual burden of HAV in the Netherlands (in DALYs per year) for acute infections occurring in the years 2000 (upper panel) and 2030 (lower panel) is shown, according to three model variants: (**i**) dynamic demography and cohort effect; (**ii**) steady state demography and cohort effect, (**iii**) dynamic demography and no cohort effect. Note the difference in scale on y-axis.

Although differences in estimated burden between dynamic compared with steady-state demographic assumptions were small for the first year of the simulation period (Figure 4), there were larger differences (fewer predicted DALYs for the static demographic model) 30 years later, seen in the age group 55–59 and older.

By subtracting the DALYs for infection in 2030 that were estimated assuming a static demography from the DALYs estimated under the baseline simulation (i.e., dynamic demography and cohort effect), and similarly the DALYs estimated from a simulation with no cohort effect (i.e., the percentage with natural immunity in 2000 was assumed constant over all age groups, at the mean value of 36.5%), the effects of population change and the replacement of immune cohorts can be separated (Figure 4). Regarding burden projections for 2030, a slightly larger impact on burden was associated with the cohort effect (a difference of 17 DALYs, compared with 12 DALYs attributed to demographic change).

**Figure 5:**
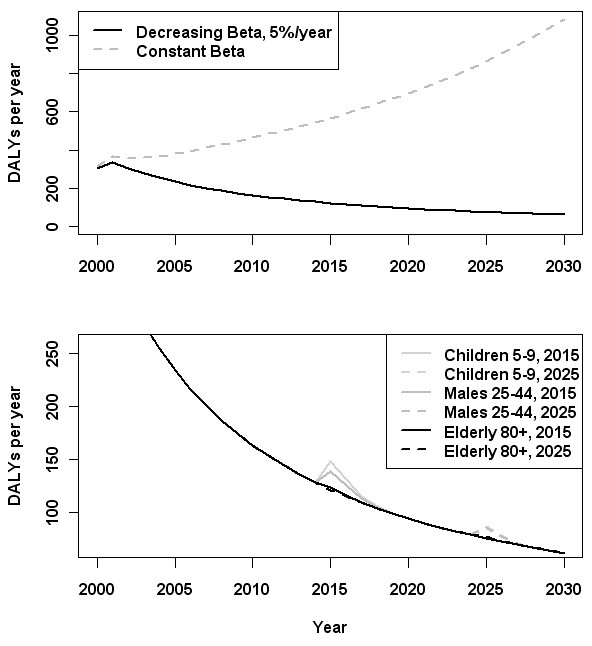
**HAV burden estimates (aggregating all age-groups) in five scenarios.** The baseline scenario, in which the transmission rate, β_a_(*t*), decreases by 5% per year in all age-groups, is compared to the situation in which β_a_ is assumed constant over time (upper panel). Three outbreak scenarios were simulated by a 3-fold greater β_a_(*t*) in the year 2015 or 2025 only, in children aged 5–9 years, men 25–44 years, and elderly persons (80+ years) (lower panel).

The results of the scenario analyses are shown in Figure 5. In Scenario 2, in which a constant transmission rate over time was assumed, there was an increase in DALYs over the simulation period, from 314 in 2000 to 1,083 in 2030. In the three outbreak scenarios, a greater total disease burden was forecast for the 5–9 years scenario (279 DALYs in 2015–16; 0.83 DALYs/100,000) than for the 25–44 year-old males scenario (e.g., 265 DALYs in 2015–16; 0.78 DALYs/100,000) or the 80+ scenario (239 DALYs in 2015–16; 0.71 DALYs/100,000). The same pattern was observed, although with lower predicted disease burdens, if the outbreak was simulated in 2025.

## Discussion

The modelled burden of HAV in the Netherlands is projected to drop in the coming decades, from 310 DALYs per year in 2000 to about one-fifth this number in 2030. This is attributable to the exponentially decreasing FOI trend assumed in the baseline simulation. However, population ageing and consequent replacement of immune birth cohorts by cohorts with low natural immunity, and the relative increase of the size of the elderly population (for whom the risk of developing symptomatic infection and associated morbidity and mortality is greatest), had a compensatory effect with respect to the estimated future burden. A greater proportion of new HAV infections, and a consequent relatively stable level of DALYs over time, was projected for the older (55+ years) age-groups.

Incorporation of a realistic model of demographic change was influential. The difference between assumptions of a static and dynamic demography with respect to incidence was visible in the 55+ years age-groups. The age distribution of new infections in 2030 was shifted from persons aged 55+ years to the <55 years age groups in the static model variant, because there is a larger proportion of the population represented by those under 55 years in the static compared with the dynamic demographic model. Consistent with this, in the dynamic demography (baseline) model the drop in DALYs forecast for 2030 was mostly localised to persons under 55 years of age.

The greater total burden forecast for the dynamic compared with the static demography is due to the increasing proportion of the population represented by the elderly over time, and to the loss of natural immunity in the older cohorts. Through comparison of the projected total disease burden in the baseline simulation with the results of the simulation with no cohort effect (in which the initial proportion immune was assumed constant across all age-groups), the cohort effect had a slightly larger impact on the estimated disease burden for infections occurring in 2030 than did the ageing and growth of the population.

This baseline scenario assumed a steadily decreasing FOI over the simulation period, in which the decline in FOI associated with improvements in socio-economic conditions following the end of the Second World War was assumed to continue to drop at the rate of 5% per year. If a constant FOI over time is assumed instead (Scenario 2), then an increasing estimated future burden between 2000 and 2030 is forecast (Figure 5).

The impact of the projected increase in population-wide susceptibility due to the loss of immune cohorts was explored in three large outbreak scenarios. Simulation of an outbreak among the elderly (80+ years) had the least impact in terms of burden in the total population (239 DALYs in 2015–16). An outbreak in men aged 25–44 years was predicted to result in a greater total disease burden (265 DALYs in 2015–16), but a simulated outbreak in primary school-aged children aged 5–9 years was associated with the largest estimated burden (279 DALYs in 2015–16). This was not merely due to the relative size of the population represented by this age-group, as indicated by the DALYs per 100,000 measure.

Previous studies of the transmission dynamics of HAV infection have accounted for the cohort/natural immunity replacement effect [18,28]; although these studies used realistic age-structured models fitted to seroprevalence data, they have assumed a steady-state demography with predictions for population growth achieved via rescaling [28]; population ageing was not taken into account. Strengths of our study are the simulation of demographic change and the estimation of the separate contributions from ageing and the replacement of naturally immune cohorts to the projected burden of disease.

There are several limitations to the current study. The first concerns how realistically the model represents the current epidemiological situation for HAV in the Netherlands. We assumed that all immunity in the population is naturally acquired; the current evidence points to a moderate proportion of immunity due to vaccination (recommended to travellers since 1994); 12.6% of the Pienter 2005/2006 survey participants had been vaccinated against HAV [1]. Vaccination is also recommended for high-risk target groups, namely patients with chronic liver disease and Turkish and Moroccan children before travelling to their country of origin. MSM are offered an HBV vaccination and often choose to be vaccinated for HAV as well. We also assumed a fixed transmission rate for travel-related infection across age-groups and time, which is a clear simplification; the oldest age-groups may have a lower likelihood of travelling to endemic countries, and travel-related transmission may change over time due to changes in the frequency of travel to, and vaccination coverage in, destination countries with endemic HAV. Thus, modelled incidence may be too low if in the future there is an increased rate of travel to endemic countries, and/or prevalence in these countries does not improve. In recent years there has been a marked rise in the proportion of imported cases (between 31 and 51% of all notified infections in the period 2007–2010 were acquired outwith the Netherlands [29]).

A second limitation concerns the adequacy of the transmission and disease burden models. Age-specific contact patterns were not incorporated, meaning that homogenous mixing between age-groups was assumed, a strong simplification. However, this should not be an issue, as our main goal was to estimate the HAV disease burden over time. The proportion of babies born immune was fixed instead of being dependent on the number of women of child-bearing age; however, because of the relatively rapid loss of maternal immunity this simplification has a minimal effect. We used a weighted average of disability weights according to the estimated overall distribution of acute illness severity [7], and the same distribution was assumed for all age groups due to a lack of relevant data. We have also not modelled interventions such as liver transplantation which would reduce the number of fatal cases and thus the YLL component of DALYs.

Third, we have not attempted to simulate the effects of control measures that would come into force should an outbreak be detected, such as the vaccination of contacts. Our simulations thus represent the extreme situation in which no intervention takes place.

The scenario in which a constant FOI over time was simulated is perhaps unrealistic in the context of the widespread availability of vaccination and effective public health education. This scenario was useful, nevertheless, for illustrating how, in the absence of reduction in the transmission rate, the combination of demographic change and the replacement of naturally immune cohorts can predict a rising disease burden. However, our baseline scenario in which we assumed a 5% annual decline in the FOI was realistic; there is evidence that HAV incidence in the Netherlands, although relatively stable from the mid-1970s to mid-1990s [26] is now steadily decreasing. Data on acute HAV cases retrieved from the Dutch national notification system indicate a decrease in HAV notifications between 1995 and 2005, from 6.5 to 1.3 per 100,000 [9]. This likely reflects a combination of a reduction in importation (affecting the proportion of travel-related cases, model parameter φ), possibly attributable to improved vaccination rates among travellers or to reduced endemicity in the destination countries (affecting the transmission rate β_trav_) [30].

## Conclusions

Universal or targetted HAV vaccination is currently not economically favourable in the Netherlands [31] due to the relatively low current disease burden and the high cost of implemention. Our study suggests that vaccination may need to be re-evaluated in a few decades’ time, when a large proportion of the population will consist of susceptible elderly persons – the age-group with the greatest risk of developing severe complications following infection. As a means of reducing the future disease burden, continuing the vaccination of travellers to endemic countries and of contacts of infected cases is essential. We suggest that economic evaluations can benefit from combining models of infection dynamics and demographic change when forecasting the future disease burden. The current approach may also be useful for estimating the burden for other infectious diseases as herpes zoster, invasive pneumococcal disease, and pertussis, especially in an ageing population.

## Abbreviations

HAV: Hepatitis A virus; MSM: Men who have sex with men; FOI: Force of infection; MSIR: Maternal immunity – Susceptible – Infectious - Recovered; BCoDE: Burden of Communicable Diseases in Europe; CI: Confidence interval; CBS: Statistics Netherlands; DALY: Disability-adjusted life-years; YLD: Years of life lost due to disability; YLL: Years of life lost

## Competing interests

The authors declare that they have no competing interests.

## Authors’ contributions

SM conceptualised the study, carried out the simulations and burden computations, and drafted the manuscript. MK conceptualised the study, advised on model design and parameterisation, interpreted the results, and edited the manuscript. M-JM, AS and EC interpreted the results and edited the manuscript. All authors read and approved the final manuscript.

## Pre-publication history

The pre-publication history for this paper can be accessed here:

http://www.biomedcentral.com/1471-2334/13/120/prepub

## Supplementary Material

Additional file 1: Figure A1 Outcome tree for hepatitis A. **Figure A2.** Acute hepatitis A cases (from notified case data, corrected for under-reporting/under-ascertainment using a multiplication factor range of 3.7-5.6 [7], and averaged over the period 2000–2010), and model predictions for the same period. Bars indicate 95% confidence intervals, derived using Latin hypercube sampling. **Table A1.** HAV disease progression model parameters [7,17,19,22]. Click here for file
