# Supplementary material for: Effects of an ageing population and the replacement of immune birth cohorts on the burden of hepatitis A in the Netherlands
Source: BMC Infect Dis. 2013 Mar 5;13:120. doi: 10.1186/1471-2334-13-120 (PMC3637296; doi:10.1186/1471-2334-13-120)
Supplement: Additional file 1: Figure A1 — Outcome tree for hepatitis A. Figure A2. Acute hepatitis A cases (from notified case data, corrected for under-reporting/under-ascertainment using a multiplication factor range of 3.7-5.6 [7], and averaged over the period 2000–2010), and model predictions for the same period. Bars indicate 95% confidence intervals, derived using Latin hypercube sampling. Table A1. HAV disease progression model parameters [7,17,19,22]. [file 1471-2334-13-120-S1.doc]

**Additional file 1**

Effects of an ageing population and the replacement of immune birth cohorts on the burden of hepatitis A in the Netherlands

Scott A McDonald, Marie-Josée J Mangen, Anita Suijkerbuijk, Edoardo Colzani and Mirjam EE Kretzschmar

BMC Infectious Diseases 2013, 13:120 http://www.biomedcentral.com/1471-2334/13/120

**Figure A1.** Outcome tree for hepatitis A.

**Symptomatic infection**

**Asymptomatic**

**R**

**R**

**Death**

**Figure A2.** Acute hepatitis A cases (from notified case data, corrected for under-reporting/under-ascertainment using a multiplication factor range of 3.7-5.6 , and averaged over the period 2000-2010), and model predictions for the same period. Bars indicate 95% confidence intervals, derived using Latin hypercube sampling.


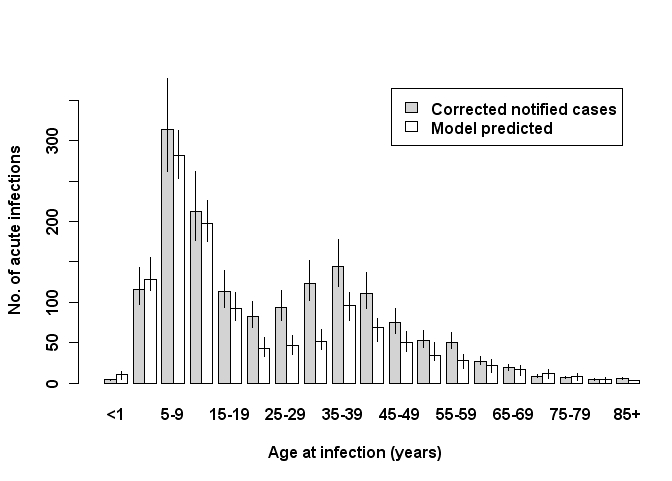


**Table A1.** HAV disease progression model parameters

| **Health outcome** | **Transitional probability** | **Disability weight** | **Duration (years)** |
| --- | --- | --- | --- |
| Acute hepatitis | -- | 0.050* | 0.082** |
| Death (age group-specific) | 0.003 (<5 yrs)† | -- | -- |
|  | 0.0018 (5–14)† |  |  |
|  | 0.0018 (15–29) § |  |  |
|  | 0.0021 (30–39) § |  |  |
|  | 0.0036 (40–49) § |  |  |
|  | 0.0081 (50–59) § |  |  |
|  | 0.0149 (60–69) § |  |  |
|  | 0.0283 (70–79) § |  |  |
|  | 0.0385 (80+) § |  |  |

* Disability weight was estimated as the weighted average of the disability weights associated with three levels of severity (hospitalisation, visiting a GP, and not visiting a GP; see Havelaar et al. [7]).

** 30-day duration of acute illness taken from Haagsma et al. [22]

† Age-dependent case-fatality rates were taken from Bauch et al. [19]

§ Age-dependent case-fatality rates were taken from Jacobs et al. [17]
